# Developmental Trauma as a Prognostic Factor for Later Psychotic Disorder in an Adolescent Clinical Cohort: A 20‐Year Follow Up Study

**DOI:** 10.1111/acps.70122

**Published:** 2026-07-01

**Authors:** Valentina Kieseppä, Covadonga M. Díaz‐Caneja, Bart P. F. Rutten, Pirkko Riipinen, Sinan Gülöksüz, Ian Kelleher

**Affiliations:** ^1^ Institute for Neuroscience and Cardiovascular Research, University of Edinburgh Edinburgh UK; ^2^ Research Unit of Clinical Medicine, Faculty of Medicine University of Oulu Oulu Finland; ^3^ Department of Child and Adolescent Psychiatry, Institute of Psychiatry and Mental Health, Hospital General Universitario Gregorio Marañón, Instituto de Investigación Sanitaria Gregorio Marañón (IiSGM), CIBERSAM, ISCIII, School of Medicine Universidad Complutense Madrid Spain; ^4^ Department of Psychiatry and Neuropsychology, School for Mental Health and Neuroscience Maastricht University Medical Centre Maastricht the Netherlands; ^5^ Department of Psychiatry Oulu University Hospital Oulu Finland; ^6^ Department of Psychiatry University of British Columbia Vancouver Canada; ^7^ Institute of Mental Health University of British Columbia Vancouver Canada; ^8^ Northern Medical Program, Division of Medical Sciences University of Northern British Columbia Prince George Canada; ^9^ Department of Psychiatry Yale School of Medicine New Haven Connecticut USA; ^10^ School of Medicine University College Dublin Dublin Ireland; ^11^ St John of God Hospitaller Services Group Hospitaller House Dublin Ireland

**Keywords:** childhood physical abuse, childhood sexual abuse, psychosis, transition, trauma

## Abstract

**Background and Hypothesis:**

Recent studies demonstrate that individuals who attend psychiatric services in adolescence, especially inpatient care, have an increased risk of psychotic disorders in adulthood. Given the extensive literature demonstrating a relationship between developmental trauma and psychosis, we investigated whether trauma history would help to identify elevated psychosis risk within a clinical cohort.

**Study Design:**

The sample consisted of patients admitted to a regional adolescent inpatient psychiatric unit (Oulu, Finland) between April 2001 and March 2006. The Kiddie Schedule for Affective Disorders and Schizophrenia was used to assess history of developmental trauma. Primary analyses investigated childhood sexual and/or physical abuse and secondary analyses investigated other types of traumatic events (car accident, other accident, fire, witness of a disaster, witness of a violent crime, victim of a violent crime, confronted with traumatic news, witness to domestic violence, other). Diagnoses from specialist healthcare were followed up in the national healthcare register until June 2023. Logistic regression was used to assess the relationship between childhood trauma and subsequent schizophrenia‐spectrum disorders (SSDs).

**Study Results:**

Of 404 adolescent inpatients admitted with non‐psychotic mental disorders, 14% reported a history of childhood sexual abuse and 27% reported a history of childhood physical abuse. Exposure to childhood sexual or physical abuse was not associated with a subsequently increased risk of SSDs (adjusted OR = 1.05, 95% CI = 0.59–1.83). Similarly, none of the other developmental adversities were associated with a subsequently increased risk of SSDs.

**Conclusions:**

In a clinical cohort made up of non‐psychotic adolescent psychiatry inpatients, a group known to be at elevated risk of psychosis, none of the assessed developmental adversities were prognostic factors for subsequent psychotic disorders.

## Summary

- Significant outcomes
  ○ In contrast to findings from general population studies, developmental trauma was not associated with future SSDs in this high‐risk cohort of psychiatric adolescent inpatients.
  ○ Factors associated with SSDs in the general population do not necessarily predict SSDs in an already high‐risk clinical population.
  ○ Our results highlight the need to investigate whether other factors might help to identify individuals at greatest risk of future psychosis in psychiatric inpatient settings.
- Limitations
  ○ Traumatic experiences were assessed retrospectively, which may have introduced recall bias.
  ○ Diagnoses of SSDs were derived from a register of public specialized health care. If there were any SSDs which were not diagnosed in public health care, these would not have been captured.

## 1 Introduction

Schizophrenia‐spectrum disorders carry a heavy burden for individuals and society, resulting in substantial functional impairment, high healthcare costs, and a mortality gap of over 10 years [1, 2, 3]. There has, accordingly, been a strong emphasis on psychosis prediction and prevention [4, 5, 6]. Recent studies have demonstrated that young people admitted to adolescent psychiatry inpatient units (for any reason) are a high‐risk group for later psychotic disorder [7, 8]. In Finland, Lång et al. [7] found that approximately 20% of individuals with a history of adolescent psychiatry inpatient admission were diagnosed with a psychotic disorder before age 30 [7]. Similarly, in the UK, O'Hare et al. [8] found that approximately 14% of individuals with a history of adolescent psychiatry inpatient admission were diagnosed with a psychotic disorder by age 32 [8]. These findings demonstrate that adolescent psychiatry inpatients are an important risk group for psychotic disorders and highlight the need for additional research to identify prognostic factors for psychosis within these clinical cohorts.

One prominent factor implicated in the development of psychosis is developmental trauma; that is, exposure to adversity during childhood [9, 10, 11, 12, 13, 14]. Certain forms of trauma, especially childhood sexual abuse (CSA) and childhood physical abuse (CPA), have been identified as prognostic factors for later diagnosis of psychotic disorder in general population studies [15, 16, 17]. Much of the existing evidence, however, relies upon retrospective reports from individuals already diagnosed with psychotic disorders, which introduces recall bias, or focuses on perceptual and ideational disturbance or “psychotic experiences”, rather than psychotic disorders [18]. Whether information on developmental trauma is a prognostic factor for later psychotic disorder in clinical populations is unknown.

In the current study, we examined whether developmental trauma was a prognostic factor for later psychotic disorder in adolescent psychiatry patients. This study recruited adolescents admitted to Oulu University Hospital's adolescent psychiatry ward, a regional inpatient ward serving the two northernmost provinces of Finland, which cover 43% of the area of Finland [19], between the years 2001 and 2006. After their admission, the patients' health care use and diagnoses were followed up using national healthcare register data up to June 2023.

### 1.1 Aims of the Study

Our primary hypothesis was that exposure to sexual abuse or physical abuse in childhood would be a prognostic factor for subsequent psychotic disorders in adulthood. Secondary (exploratory) analyses investigated the relationship between other types of adversity, including exposure to/involvement in car accidents, other accidents, fire, natural disasters, violent crime, distressing news, domestic violence and “other” potential traumas, and risk of subsequent psychotic disorder.

## 2 Methods

### 2.1 Study Sample

All new adolescent (age 13–17) patients (*n* = 637) admitted to Oulu University Hospital, Department of Psychiatry inpatient ward between April 2001 and March 2006 (hereafter referred as index hospitalization), were invited to participate [19]. Exclusion criteria included (1) aged over 18 years (*n* = 1), (2) diagnosis of an intellectual disability (*n* = 26), or (3) diagnosis of an organic brain disorder (*n* = 3). In addition, patients were excluded if the inpatient stay was too short for the interviews to be completed (*n* = 22). The 77 individuals or their guardians declined to give consent to participate. Overall, out of 607 eligible adolescents, 508 (83.7%) participated. For the current study, individuals who had already been diagnosed with psychosis at the time of the assessment were excluded, resulting in a final sample of 404 participants.

Written informed consent for participation in the study was obtained from all the study participants and their legal guardians. The project has been approved by the Ethics Committee of Oulu University Hospital. Permission to use the personal register data has been obtained from the Finnish Social and Health Data Permit Authority Findata (THL/4612/14.02.00/2022).

### 2.2 Developmental Trauma

During the inpatient admission, adolescents were assessed using the Kiddie Schedule for Affective Disorders and Schizophrenia for School‐Age Children—Present and Lifetime (K‐SADS‐PL) [20]. The interviews were conducted by trained research assistants under the supervision of a psychiatrist or, in some cases, by the treating psychiatrist. For the current study, responses to the post‐traumatic stress disorder screening section were used. The section inquires whether the participant has ever in their lifetime experienced any of the following traumatic events: (a) *car accident*, (b) *other accident*, (c) *fire*, (d) *witness of a disaster*, (e) *witness of a violent crime*, (f) *victim of a violent crime*, (g) *confronted with traumatic news*, (h) *witness to domestic violence*, (i) *physical abuse*, (j) *sexual abuse*, or (k) *other*. Each item is scored either *yes* or *no*. The severity and potential recurrence of the trauma exposure were not recorded. This study uses information derived directly from the participant.

Our main exposure variables of interest were childhood *physical abuse* (CPA) and *sexual abuse* (CSA). They were combined into CPA or CSA to take value *yes* if participants reported experiencing either and to otherwise take value *no*. In addition, a variable *any trauma* was formed of all the inquired trauma types to indicate whether the participant had ever experienced any of the inquired traumatic events.

### 2.3 Registered Diagnoses of Schizophrenia‐Spectrum Disorders

We retrieved information on visits and hospital admissions in public specialized health care from the national Finnish Care Register for Health Care (FCRHC) register, which is considered highly reliable for research purposes [21], including for diagnoses of schizophrenia [22]. It captures all specialized health care outpatient visits, inpatient admissions and associated diagnoses. Information on the date of the visit or admission and the associated diagnoses was retrieved. Information on inpatient treatments was available for the whole lifetime of participants, while data on outpatient visits to specialized health care was available from the year 1998 onwards. Both outpatient and inpatient data were available until June 2023. In Finland, SSDs are almost always treated within specialized health care.

Our outcome of interest was schizophrenia‐spectrum disorders (SSDs). A participant was categorized to have been diagnosed with an SSD if they had either a visit or an admission with primary or secondary diagnosis of F20–F29 (based on the 10th revision of the International Statistical Classification of Diseases and Related Health Problems (ICD‐10), which has been used in Finland since 1996).

All individuals who had a registered diagnosis of an SSD before or at their discharge date from the index hospitalization were excluded (*n* = 95). We also ensured that there were no inpatient admissions with an SSD prior to 1996 with diagnostic codes (1) 295 Schizophrenia, (2) 297 Delusional disorder, or (3) 298 Other psychotic disorder based on the 9th revision of the International Statistical Classification of Diseases and Related Health Problems (ICD‐9). In addition, nine individuals did not have an SSD diagnosis in the health care register before or at their discharge date from index hospitalization but had a validated SSD assessed with the K‐SADS‐PL diagnostic interview. These individuals were also excluded, resulting in a final sample of 404 participants.

### 2.4 Covariates

Information on *sex* and *age at admission* was derived from initial study records. *Psychiatric symptom severity* scores were calculated based on K‐SADS‐PL by taking the mean of the present and lifetime ratings in each of the subsections similarly as in [23], with a few exceptions: substance use and alcohol use scores were calculated based on K‐SADS‐PL mean scores on alcohol abuse and substance abuse screening sections. The following symptom categories were used: *alcohol abuse symptoms*, *substance abuse symptoms*, *depression symptoms*, *mania symptoms*, *psychosis symptoms*, *anxiety symptoms*, *eating disorder symptoms* and *oppositional defiant disorder/conduct disorder symptoms*.

In addition to K‐SADS‐PL, participants were also interviewed with the Addiction Severity Index (EuropASI) [24]. Information on patients' *living arrangement, learning difficulties* (yes/no), *parental mental health problems*, and *parental occupation* were derived from EuropASI. The variable *living arrangements* was based on reported living arrangements during the past year (or, if missing, reported current living arrangements) and was categorized into the following categories: living with (1) two parents, (2) single parent, or (3) foster care, institution or other (e.g., living alone or with other relatives). The variable *parental mental health problems* was based on participants' evaluation of whether their mother or father had ever had substance use or mental health problems which, in the opinion of the participant, would have required treatment (yes/no). *Parental occupation* was categorized as: (1) not in employment, (2) blue collar worker, (3) lower white‐collar worker, (4) upper white‐collar worker/employer/entrepreneur. If occupations of both parents were reported, the highest (based on the order described above) was used.

There was a relatively high number of missing values on the variables used to measure parental information. Because the missingness is unlikely to be random (participants who cannot report information on their parents might be more likely not to be in close contact with their parents), instead of excluding participants with missing values, for each of the parental variables, we created a category “missing”. This allowed us to keep individuals with missing information on parental variables in the models, rather than excluding them and potentially introducing bias due to non‐random missingness.

### 2.5 Statistical Analysis

Table 1 presents the descriptive statistics of the sample. Cumulative incidence of diagnoses of SSDs after discharge was calculated for having experienced either CSA or CPA, and the prevalence of CSA or CPA was calculated separately for those who eventually received a diagnosis of an SSD and for those who did not. As secondary analyses, similar figures were calculated for all the other traumatic events of interest.

**TABLE 1:** Descriptive statistics.

Sex (female) (*n* (%))	244 (60%)
Age (mean (SD))	15.4 (1.3)
Duration of admission in days (mean (SD))	16.5 (30.9)
Validated diagnosis at baseline (*n*, %)	
Psychotic disorder	0 (0%)
Anxiety disorder	100 (25%)
Affective disorder	227 (56%)
Substance use disorder	169 (42%)
Conduct disorder	199 (49%)
Other disorder	38 (9%)
Traumatic experiences (*n*, %)	
Physical or sexual abuse	144 (36%)
Sexual abuse	56 (14%)
Physical abuse	109 (27%)
Car accident	42 (10%)
Other accident	26 (6%)
Fire	13 (3%)
Witness of disaster	5 (1%)
Witness of a violent crime	90 (22%)
Victim of a violent crime	69 (17%)
Traumatic News	186 (46%)
Witness to domestic violence	121 (30%)
Other trauma	36 (9%)
Any trauma	300 (74%)
Learning difficulties (*n*, %)	142 (35%)
Living arrangements	
Two‐parent household	204 (51%)
Single‐parent household	86 (21%)
Foster care, institution or other	111 (28%)
Parental mental health problems (*n*, %)	
Yes	164 (41%)
No	189 (47%)
Missing	51 (13%)
Parental occupation (*n*, %)	
Not in employment	48 (12%)
Blue collar worker	201 (50%)
Lower white‐collar worker	34 (8%)
Upper white‐collar, employer or entrepreneur	105 (26%)
Missing	16 (4%)

Diagnosis of an SSD in specialized health care after discharge from index hospitalization was assessed using logistic regression. The main independent variable was experience of CSA or CPA. Statistical significance was set at *p* < 0.05. We adjusted for sex, age at admission, symptom severity of different K‐SADS‐PL measured domains, reported learning difficulties, living arrangements, parental mental health problems, and parental occupation. As secondary analyses, similar models were run for each of the other traumatic events. We also carried out post hoc exploratory analyses to investigate (1) whether interpersonal trauma (defined as CSA, CPA, exposure to domestic violence, or victim of a violent crime) was associated with subsequent risk of SSD and (2) whether the number of reported traumatic experience types, as a proxy for severity, was associated with subsequent risk of SSD (Table S1). Both unadjusted and adjusted estimates were produced. A Bonferroni correction was applied to the *p*‐values of the secondary analyses to account for multiple testing, leading to a statistical significance level of *p* < 0.0045.

Sex‐stratified models are available in Tables S2 and S3. To account for the possibility that the effects of CSA/CPA on SSD risk could be mediated by psychotic symptoms at index admission, as a sensitivity analysis, we repeated the main analysis without controlling for psychotic symptoms (Table S4).

## 3 Results

Descriptive statistics are shown in Table 1.

A higher percentage of the sample was female (60%). The most prevalent diagnoses at baseline were affective disorders (56%), conduct disorders (49%), and substance use disorders (42%). Overall, a high percentage (75%) of the sample had experienced at least one traumatic event. In total, 144 individuals had a history of CPA (*n* = 109) and/or CSA (*n* = 56).

In total, 91 individuals (23% of the sample) went on to be diagnosed with an SSD by the end of follow‐up. CSA or CPA were reported by 38% of individuals who went on to develop an SSD, compared to 36% of individuals who did not (Table 2). The cumulative incidence of an SSD did not differ between those who reported CSA or CPA and those who did not: among those who reported CSA or CPA, the cumulative incidence of SSD was 24% compared to 22% among those who did not report CSA or CPA.

**TABLE 2:** Diagnosis of an SSD in specialized health care between discharge and July 2023 and trauma exposure.

	Prevalence of reported trauma at baseline		Cumulative incidence of SSD	
	Diagnosed SSD	No SSD diagnosed	Reported traumatic experience	Traumatic experience not reported
Main analysis				
Physical or sexual abuse	34 (38%)	110 (36%)	34 (24%)	55 (22%)
Secondary analyses				
Physical abuse	25 (28%)	84 (27%)	25 (23%)	65 (22%)
Sexual abuse	14 (16%)	42 (14%)	14 (25%)	75 (22%)
Car accident	7 (8%)	35 (11%)	7 (17%)	83 (23%)
Other accident	6 (7%)	20 (6%)	6 (23%)	84 (22%)
Fire	5 (6%)	8 (3%)	5 (38%)	85 (22%)
Witness of a disaster	< 5	< 5	< 5	88 (22%)
Witness of a violent crime	27 (30%)	63 (20%)	27 (30%)	63 (20%)
Victim of a violent crime	20 (23%)	49 (16%)	20 (29%)	69 (21%)
Traumatic News	48 (53%)	138 (45%)	48 (26%)	42 (20%)
Domestic violence	26 (30%)	95 (31%)	26 (21%)	62 (23%)
Other trauma	8 (9%)	28 (9.4%)	8 (22%)	79 (23%)
Any trauma	78 (86%)	222 (72%)	78 (26%)	13 (13%)

*Note:* All individuals with a diagnosis of an SSD before or at their discharge date are excluded.

Out of those who later received a diagnosis of an SSD, 86% had experienced at least some type of traumatic event, but most of those who did not receive a diagnosis of an SSD had also experienced at least one traumatic event (72%).

Table 3 shows the results of logistic regression assessing risk for future SSDs with CSA or CPA. A report of CSA or CPA was not a prognostic factor for future SSDs in the unadjusted model (OR = 1.11, 95% CI = 0.86–1.80) or in the adjusted model (OR = 1.05, 95% CI = 0.60–1.84).

**TABLE 3:** Results of the main analysis.

	OR (95% CI)	*p*
Unadjusted model		
Physical or sexual abuse	1.11 (0.68–1.80)	0.682
Adjusted model		
Physical or sexual abuse	1.05 (0.60–1.84)	0.854
Age at admission	1.20 (0.97–1.5)	0.098
Sex (ref. Male)	0.58 (0.31–1.10)	0.096
Symptoms measured by K‐SADS‐PL		
Depression symptoms	0.82 (0.34–1.93)	0.656
Mania symptoms	0.73 (0.19–2.41)	0.628
Psychosis symptoms	1.90 (0.71–5.00)	0.195
Anxiety symptoms	1.65 (0.4–6.72)	0.484
Alcohol consumption	1.46 (0.41–5.13)	0.553
Substance consumption	1.87 (0.35–9.82)	0.459
Eating disorder symptoms	2.12 (0.33–12.32)	0.405
Conduct disorder symptoms	1.03 (0.40–2.64)	0.949
Learning difficulties (ref. No)	2.21 (1.31–3.76)	**0.003**
Living arrangements (ref. Living with two parents)		
Foster care/institution/other	1.05 (0.51–2.12)	0.890
Single‐parent household	0.84 (0.41–1.67)	0.627
Parental psychiatric or substance abuse problems (ref. No)		
Yes	0.98 (0.54–1.77)	0.950
Missing	1.2 (0.53–2.65)	0.651
Parental occupation (ref. Upper white collar/Employer/Entrepreneur)		
Not in employment	2.02 (0.84–4.83)	0.114
Blue collar worker	1.34 (0.70–2.65)	0.393
Lower white‐collar worker	2.66 (0.95–7.23)	0.057
Missing	1.23 (0.29–4.57)	0.764

*Note:* The unadjusted and the full adjusted logistic regression model is reported (In the adjusted model, 16 observations were dropped due to missingness). Bold values indicate significance level of *p* < 0.05.

Abbreviations: CI = confidence interval; OR = odds ratio.

Table 4 shows the results of the secondary analyses. None of the other reported traumatic events were prognostic factors for later SSDs in the Bonferroni corrected analyses.

**TABLE 4:** Results of the secondary analyses.

	Unadjusted models			Adjusted models		
	OR (95% CI)	Uncorrected *p*	Bonferroni corrected *p*	aOR (95% CI)	Uncorrected *p*	Bonferroni corrected *p*
Physical abuse	1.03 (0.60–1.73)	0.911	1.00	0.89 (0.48–1.61)	0.703	1.00
Sexual abuse	1.16 (0.59–2.20)	0.650	1.00	1.20 (0.55–2.52)	0.640	1.00
Car accident	0.67 (0.26–1.47)	0.346	1.00	0.30 (0.10–0.78)	**0.021**	0.228
Other accident	1.04 (0.37–2.53)	0.936	1.00	0.86 (0.28–2.33)	0.776	1.00
Fire	2.23 (0.66–6.85)	0.170	1.00	1.90 (0.51–6.48)	0.315	1.00
Witness of a crime	1.68 (0.98–2.83)	0.055	0.599	1.07 (0.55–2.05)	0.837	1.00
Victim of a crime	1.54 (0.85–2.74)	0.145	1.00	0.80 (0.38–1.63)	0.552	1.00
Traumatic News	1.42 (0.89–2.29)	0.141	1.00	1.06 (0.62–1.81)	0.830	1.00
Domestic violence	0.93 (0.55–1.55)	0.788	1.00	0.84 (0.46–1.51)	0.568	1.00
Other trauma	0.98 (0.40–2.14)	0.955	1.00	0.65 (0.23–1.62)	0.376	1.00
Any trauma	2.32 (1.27–4.57)	**0.010**	0.105	1.96 (0.98–4.18)	0.067	0.739

*Note:* Both unadjusted and adjusted estimates are reported. For the adjusted models, only estimates for the main variables of interest are reported. The uncorrected bold *p*‐values indicate a significance level of *p* < 0.05.

Abbreviations: aOR = adjusted odds ratio; CI = confidence interval; OR = odds ratio.

In post hoc exploratory analyses, we did not find a significant relationship between interpersonal violence and risk of SSD (OR = 1.43, 95% CI = 0.89–2.34; see Table S1). Similarly, in post hoc exploratory analyses, we did not find a significant relationship between exposure to increasing numbers of trauma types and risk of SSD (OR = 1.08; 95% CI = 0.93–1.24; see Table S1).

## 4 Discussion

In this 20‐year longitudinal study of adolescent psychiatric inpatients, we investigated whether developmental trauma was a prognostic factor for future SSD. As in previous research [25], there was a high risk of later psychotic disorder in adolescent inpatients when followed to adulthood, with 23% going on to be diagnosed with an SSD by the end of follow up. Also in line with previous studies in adolescent psychiatric inpatient samples [26, 27], traumatic experiences were common in our inpatient cohort. Specifically, a history of CSA or CPA was recorded for just over one third (36%) of participants—CSA for 14% and CPA for 27%. Contrary to our hypotheses, however, CSA and CPA were not associated with a significantly increased risk of later SSD. In total, 24% of adolescents who reported CSA or CPA went on to be diagnosed with an SSD by the end of study follow up. Similarly, however, 22% of individuals who did not report CSA or CPA went on to be diagnosed with an SSD.

In terms of our secondary analyses, we evaluated a large number of adverse experiences for a potential relationship with SSDs. None of the additional traumatic experiences were associated with a significantly increased risk of SSD when followed to adulthood. Similarly, in post hoc exploratory analyses, we did not find a relationship between exposure to interpersonal violence or cumulative number of trauma types and risk of SSD.

Our results appear to contrast with a substantial body of research linking childhood trauma to psychosis [9, 10, 11, 12, 13]. Several factors may explain this discrepancy, most notably differences in study design and the nature of comparison groups. Much of the existing evidence derives from general population studies or case–control designs where individuals exposed to adversity have been compared to unexposed individuals. Groups who are exposed versus not exposed to developmental trauma may systematically differ in important ways, beyond their trauma experiences, many of which could potentially affect the relationship between childhood adversity and psychosis. This includes, for example, differences in personality characteristics such as rigidity and impulsivity, history of attachment problems, anxiety, and substance use, and family factors such as lower parental education and single parent families [28, 29, 30]. Given both our exposure and comparison groups were derived from a clinical cohort, they are likely to have differed to a lesser degree than would be expected if the comparison group had been a general population sample. This finding highlights that prognostic factors at a population level do not necessarily predict the same outcomes when applied to clinical samples.

The “any trauma” variable showed a trend association for later SSD, which fell short of traditional statistical significance. The finding, however, that nearly three‐quarters of the sample endorsed at least one traumatic event means that the clinical utility of an “any trauma” variable for risk stratification is inherently limited.

In order to study the prognostic validity of trauma exposure for later SSDs, it was necessary to exclude participants who had already been diagnosed with an SSD at baseline. Evidence from retrospective studies suggests that childhood trauma exposure might be linked to an earlier age of onset of SSDs [14, 31], and our results cannot account for the possibility that some individuals who had experienced trauma would have already developed a psychotic disorder by the study baseline. However, importantly, our results suggest that exposure to CSA or CPA does not have additional prognostic value for identifying future SSDs among non‐psychotic psychiatric adolescent inpatients, a group that is at very high risk of future psychosis. Given this overall high risk, future research should continue to test whether other clinical or demographic factors might help to identify those who ultimately go on to develop an SSD [32].

Our findings do not suggest that trauma is not a prognostic factor for mental ill health more broadly. Indeed, the high prevalence of traumatic experiences in this inpatient cohort highlights a relationship between trauma and mental ill health in adolescence, albeit this finding does not necessarily point to a causal relationship. Rather, our findings point to a lack of a specific relationship between trauma and psychotic disorders, including for childhood sexual and physical abuse.

### 4.1 Strengths and Limitations

Strengths of our study include a high participation rate (83.7%), the use of a combination of clinical and register data, which allowed us to draw information from multiple sources, and a long term (> 17 year) follow‐up, with no attrition. While the K‐SADS‐PL semi‐structured interview allowed systematic assessment of traumatic experiences across the sample, we were not able to differentiate between single traumatic events and persistent trauma even though there may be a stronger relationship between persistent trauma and SSDs [33]. Persistent trauma, however, would still have been captured within our trauma variables. While our assessment of trauma occurred prior to SSD diagnosis, reducing the bias of a post‐diagnostic assessment, it was, nonetheless, a retrospective assessment, introducing risk of recall bias. Recall bias, however, would be expected to bias toward a positive finding, which was not what we found in this study. Furthermore, given that the participants were teenagers at the time of assessment, developmental trauma would have been more proximal to the time of the assessment than studies conducted in adulthood, increasing recall accuracy. Our information on diagnoses came solely from public specialist health care, which does not necessarily reflect the true incidence of SSDs in the population. There are, however, no private psychiatric hospitals in Finland. We cannot rule out that there may have been individuals who developed SSDs who did not come to the attention of clinical services.

## 5 Conclusions

In an approximately 20‐year longitudinal study looking at psychosis risk among individuals who were admitted to inpatient adolescent psychiatry services, we did not find evidence that CSA and CPA were associated with an elevated risk of psychosis. Similarly, in secondary analyses, we did not find a significant association between a range of other traumatic experiences and subsequent psychosis risk. Given the high risk of SSDs in this clinical population, future research to test other clinical and demographic prognostic factors for psychosis will be important.

## Author Contributions

I.K. and V.K. designed the study. V.K. managed the statistical analyses and wrote the first draft of the manuscript. C.M.D.‐C., B.P.F.R., P.R., S.G., and I.K. have critically revised the work.

## Funding

This work was supported by awards from the Health Research Board (ECSA‐2020‐005), the Academy of Medical Sciences (APR8\1005), the UK Department for Business, Energy and Industrial Strategy, and Horizon Europe [Youth‐GEMs, grant agreement number: 101057182]. Dr. Díaz‐Caneja has received grant support from Instituto de Salud Carlos III, Spanish Ministry of Science and Innovation (PI17/00481, PI20/00721, PI23/00625, JR19/00024) and Horizon Europe [YOUTHreach, grant agreement number: 101156514].

## Ethics Statement

The project has been approved by the Ethics Committee of Oulu University Hospital.

## Consent

Participants and their legal guardians have given their written informed consent to participate in the study.

## Conflicts of Interest

Dr. Díaz‐Caneja has received honoraria and/or travel support from Angelini, Johnson&Johnson, and Viatris, unrelated to this work. The other authors declare no conflicts of interest.

## Supporting information


**Table S1:** Post hoc analyses. Results of the logistic regression models predicting future SSDs with interpersonal trauma and number of reported trauma types.
**Table S2:** Results of the main analysis stratified by sex. The unadjusted and the full adjusted logistic regression model is reported.
**Table S3:** Results of the secondary analyses stratified by sex. Both unadjusted and adjusted estimates are reported, only for the main independent variables of interest.
**Table S4:** Results of the sensitivity analysis. Adjusted model without psychotic symptoms as a predictor.

## Data Availability

Research data are not publicly available.
