# Supplementary material for: Developmental Trauma as a Prognostic Factor for Later Psychotic Disorder in an Adolescent Clinical Cohort: A 20‐Year Follow Up Study
Source: Acta Psychiatr Scand. 2026 Jul 1;154(5):352–60. doi: 10.1111/acps.70122 (PMC13630490; doi:10.1111/acps.70122)
Supplement: Supplementary file 1 [file ACPS-154-352-s001.docx]

**SUPPLEMENTARY MATERIAL**

| Supplementary Table 1. Post-hoc analyses. Results of the logistic regression models predicting future SSDs with interpersonal trauma (Model 1) and number of reported trauma types (Model 2). Both unadjusted estimates and estimates adjusted for covariates are reported. | | | | | | | | |
| --- | --- | --- | --- | --- | --- | --- | --- | --- |
|  | Unadjusted | | | | Adjusted | | | |
|  | OR | Lower 95% CI | Upper  95%  CI | p value | AdjustedOR | Lower 95%  CI | Upper  95%  CI | p value |
| Model 1 |  |  |  |  |  |  |  |  |
| Interpersonal trauma | 1.43 | 0.89 | 2.34 | 0.148 | 1.31 | 0.75 | 2.30 | 0.351 |
| Model 2 |  |  |  |  |  |  |  |  |
| Number of reported trauma types | 1.08 | 0.93 | 1.24 | 0.303 | 0.92 | 0.76 | 1.10 | 0.360 |
| Note. OR = odds ratio; CI = confidence interval. | | | | | | | | |

| Supplementary Table 2. Results of the main analysis stratified by sex. The unadjusted and the full adjusted logistic regression model is reported. | | | | | | | | |
| --- | --- | --- | --- | --- | --- | --- | --- | --- |
|  | Males | | | | Females | | | |
|  | OR | Lower 95% CI | Upper  95%  CI | p value | OR | Lower 95%  CI | Upper  95%  CI | p value |
| **Unadjusted model** |  |  |  |  |  |  |  |  |
| Physical or sexual abuse | 1.01 | 0.45 | 2.18 | 0.977 | 1.35 | 0.70 | 2.57 | 0.367 |
| **Adjusted model** |  |  |  |  |  |  |  |  |
| Physical or sexual abuse | 0.83 | 0.31 | 2.11 | 0.702 | 1.33 | 0.59 | 3.02 | 0.490 |
| Age at admission | 1.27 | 0.90 | 1.84 | 0.183 | 1.07 | 0.78 | 1.46 | 0.692 |
| Symptoms measured by K-SADS |  |  |  |  |  |  |  |  |
| Depression symptoms | 1.40 | 0.31 | 6.24 | 0.656 | 0.83 | 0.25 | 2.62 | 0.759 |
| Mania symptoms | 0.64 | 0.06 | 4.73 | 0.69 | 0.75 | 0.12 | 3.78 | 0.737 |
| Psychosis symptoms | 4.81 | 0.76 | 33.01 | 0.099 | 1.04 | 0.27 | 3.67 | 0.953 |
| Anxiety symptoms | 0.93 | 0.10 | 9.38 | 0.951 | 2.16 | 0.26 | 16.54 | 0.460 |
| Alcohol consumption | 7.63 | 0.98 | 65.14 | 0.056 | 0.3 | 0.05 | 1.86 | 0.205 |
| Substance consumption | 0.33 | 0.02 | 4.16 | 0.407 | 10.5 | 0.61 | 219.37 | 0.112 |
| Eating disorder symptoms |  |  |  |  | 2.34 | 0.31 | 16.05 | 0.389 |
| Conduct disorder symptoms | 1.47 | 0.39 | 5.59 | 0.565 | 0.64 | 0.11 | 3.36 | 0.598 |
| Learning difficulties (ref. No) | 1.64 | 0.71 | 3.86 | 0.245 | 3.4 | 1.61 | 7.33 | **0.001** |
| Living arrangements (ref. Living with two parents) |  |  |  |  |  |  |  |  |
| Foster care/institution/other | 1.11 | 0.31 | 3.96 | 0.873 | 1.31 | 0.50 | 3.35 | 0.569 |
| Single-parent household | 1.15 | 0.38 | 3.42 | 0.804 | 0.90 | 0.29 | 2.50 | 0.839 |
| Parental psychiatric or substance abuse problems (ref. No) |  |  |  |  |  |  |  |  |
| Yes | 0.35 | 0.11 | 1.05 | 0.068 | 1.86 | 0.86 | 4.13 | 0.118 |
| Missing | 1.24 | 0.37 | 4.05 | 0.726 | 1.06 | 0.21 | 3.98 | 0.939 |
| Parental occupation (ref. Upper white collar/Employer/Entrepreneur) |  |  |  |  |  |  |  |  |
| Not in employment | 4.41 | 1.03 | 20.55 | 0.05 | 1.50 | 0.43 | 5.05 | 0.512 |
| Blue collar worker | 2.06 | 0.71 | 6.59 | 0.197 | 1.12 | 0.45 | 2.95 | 0.813 |
| Lower white-collar worker | 3.99 | 0.81 | 20.08 | 0.086 | 2.52 | 0.54 | 10.66 | 0.214 |
| Missing | 0.95 | 0.10 | 6.37 | 0.957 | 6.65 | 0.49 | 169.11 | 0.165 |

*Note.* Because eating disorder symptoms were virtually non-existent in males, model including only males is not adjusted for eating disorder symptoms. OR = odds ratio; CI = confidence interval. Bold values indicate significance level of p < 0.05

| Supplementary Table 3. Results of the secondary analyses stratified by sex. Both unadjusted and adjusted estimates are reported, only for the main independent variables of interest. | | | | | | | | | | |
| --- | --- | --- | --- | --- | --- | --- | --- | --- | --- | --- |
|  | **Males** | | | | | | | | | |
|  | Unadjusted models | | | | | Adjusted models | | | | |
|  | OR | Lower 95% CI | Upper 95% CI | Uncorrected p value | Bonferroni corrected p value | Adjusted OR | Lower 95% CI | Upper 95% CI | Un corrected p value | Bonferroni corrected p-value |
| Physical abuse | 0.91 | 0.40 | 1.99 | 0.82 | 1.000 | 0.75 | 0.28 | 1.94 | 0.566 | 1.000 |
| Sexual abuse | 1.83 | 0.24 | 11.45 | 0.515 | 1.000 | 1.58 | 0.14 | 15.63 | 0.693 | 1.000 |
| Car accident | 0.70 | 0.22 | 1.90 | 0.512 | 1.000 | 0.17 | 0.03 | 0.67 | **0.018** | 0.185 |
| Other accident | 0.78 | 0.17 | 2.70 | 0.716 | 1.000 | 0.45 | 0.08 | 2.06 | 0.331 | 1.000 |
| Fire | 2.06 | 0.39 | 9.75 | 0.357 | 1.000 | 1.37 | 0.20 | 7.92 | 0.727 | 1.000 |
| Witness of a crime | 2.11 | 0.98 | 4.51 | 0.055 | 0.600 | 1.54 | 0.54 | 4.28 | 0.411 | 1.000 |
| Victim of a crime | 1.79 | 0.79 | 3.97 | 0.153 | 1.000 | 0.90 | 0.29 | 2.65 | 0.846 | 1.000 |
| Traumatic News | 1.36 | 0.67 | 2.77 | 0.398 | 1.000 | 1.05 | 0.45 | 2.46 | 0.903 | 1.000 |
| Domestic violence | 0.61 | 0.25 | 1.37 | 0.249 | 1.000 | 0.62 | 0.21 | 1.70 | 0.361 | 1.000 |
| Other trauma | 1.01 | 0.21 | 3.72 | 0.985 | 1.000 | 0.72 | 0.12 | 3.53 | 0.7 | 1.000 |
| Any trauma | 2.25 | 0.96 | 5.94 | 0.079 | 0.865 | 1.55 | 0.56 | 4.69 | 0.415 | 1.000 |
|  | **Females** | | | | | | | | | |
|  | Unadjusted models | | | | | Adjusted models | | | | |
|  | OR | Lower 95% CI | Upper 95% CI | Uncorrected p value | Bonferroni corrected p value | Adjusted OR | Lower 95% CI | Upper 95% CI | Uncorrected p value | Bonferroni corrected p value |
| Physical abuse | 1.16 | 0.56 | 2.30 | 0.681 | 1.000 | 0.90 | 0.36 | 2.18 | 0.826 | 1.000 |
| Sexual abuse | 1.34 | 0.62 | 2.76 | 0.445 | 1.000 | 1.33 | 0.54 | 3.15 | 0.526 | 1.000 |
| Car accident | 0.47 | 0.07 | 1.72 | 0.325 | 1.000 | 0.30 | 0.04 | 1.37 | 0.173 | 1.000 |
| Other accident | 1.28 | 0.28 | 4.37 | 0.721 | 1.000 | 1.43 | 0.25 | 6.17 | 0.657 | 1.000 |
| Fire | 2.14 | 0.29 | 11.34 | 0.387 | 1.000 | 3.02 | 0.37 | 18.99 | 0.246 | 1.000 |
| Witness of a crime | 1.27 | 0.57 | 2.66 | 0.538 | 1.000 | 0.96 | 0.33 | 2.53 | 0.929 | 1.000 |
| Victim of a crime | 1.13 | 0.43 | 2.66 | 0.79 | 1.000 | 0.77 | 0.23 | 2.24 | 0.642 | 1.000 |
| Traumatic News | 1.42 | 0.75 | 2.70 | 0.281 | 1.000 | 1.26 | 0.59 | 2.70 | 0.557 | 1.000 |
| Domestic violence | 1.30 | 0.65 | 2.52 | 0.447 | 1.000 | 0.93 | 0.41 | 2.06 | 0.864 | 1.000 |
| Other trauma | 1.01 | 0.32 | 2.67 | 0.982 | 1.000 | 0.55 | 0.14 | 1.77 | 0.35 | 1.000 |
| Any trauma | 2.50 | 1.07 | 6.87 | **0.049** | 0.540 | 3.61 | 1.22 | 12.98 | **0.031** | 0.343 |

*Note.* Because eating disorder symptoms were virtually non-existent in males, models including only males are not adjusted for eating disorder symptoms. OR = odds ratio; CI = confidence interval.

| Supplementary Table 4. Results of the sensitivity analysis. Adjusted model without psychotic symptoms as a predictor. | | | | |
| --- | --- | --- | --- | --- |
|  | OR | Lower 95% CI | Upper 95% CI | p value |
| Physical or sexual abuse | 1.06 | 0.60 | 1.85 | 0.827 |
| Age at admission | 1.20 | 0.97 | 1.49 | 0.103 |
| Sex (ref. Male) | 0.63 | 0.33 | 1.18 | 0.145 |
| Symptoms measured by K-SADS-PL |  |  |  |  |
| Depression symptoms | 0.88 | 0.37 | 2.01 | 0.757 |
| Mania symptoms | 0.74 | 0.20 | 2.40 | 0.631 |
| Anxiety symptoms | 1.86 | 0.46 | 7.52 | 0.377 |
| Alcohol consumption | 1.43 | 0.41 | 4.98 | 0.570 |
| Substance consumption | 2.77 | 0.6 | 13.21 | 0.194 |
| Eating disorder symptoms | 1.95 | 0.31 | 11.09 | 0.453 |
| Conduct disorder symptoms | 0.97 | 0.38 | 2.47 | 0.958 |
| Learning difficulties (ref. No) | 2.20 | 1.30 | 3.74 | **0.003** |
| Living arrangements (ref. Living with two parents) | |  |  |  |
| Foster care/institution/other | 1.06 | 0.52 | 2.13 | 0.873 |
| Single-parent household | 0.85 | 0.42 | 1.67 | 0.640 |
| Parental psychiatric or substance abuse problems (ref. No) | |  |  |  |
| Yes | 0.96 | 0.53 | 1.72 | 0.879 |
| Missing | 1.21 | 0.53 | 2.66 | 0.632 |
| Parental occupation (ref. Upper white collar/Employer/Entrepreneur) | | | | |
| Not in employment | 2.05 | 0.85 | 4.90 | 0.107 |
| Blue collar worker | 1.41 | 0.74 | 2.79 | 0.305 |
| Lower white-collar worker | 2.99 | 1.10 | 8.00 | **0.029** |
| Missing | 1.31 | 0.30 | 4.84 | 0.700 |
| *Note.* OR = odds ratio; CI = confidence interval. Bold values indicate significance level of p < 0.05 | | | | |
